# Supplementary material for: Celiac Disease Defined by Over-Sensitivity to Gliadin Activation and Superior Antigen Presentation of Dendritic Cells
Source: Int J Mol Sci. 2021 Sep 15;22(18):9982. doi: 10.3390/ijms22189982 (PMC8469067; doi:10.3390/ijms22189982)
Supplement: Supplementary file 1 [file ijms-22-09982-s001.zip › ijms-1342053-supplementary - Copy.pdf]

International Journal of *Molecular Sciences*

Article

Celiac Disease Defined by Over-Sensitivity to Gliadin Activation and Superior Antigen Presentation of Dendritic Cells

Michael Hudec [1,*], Kamila Riegerová [2], Jan Pala [3,4], Viera Kútna [4], Marie Černá [1] and Valerie Bríd O´Leary [1]

[1] Department of Medical Genetics, Third Faculty of Medicine, Charles University, Ruská 87, 100 00 Prague, Czech Republic; marie.cerna@lf3.cuni.cz (M.Č.); valerie.oleary@lf3.cuni.cz (V.B.O.)
[2] Department of Immunology and Clinical Biochemistry, Third Faculty of Medicine, Charles University, Ruská 87, 100 00 Prague, Czech Republic; kamila.riegerova@lf3.cuni.cz
[3] Department of Pathophysiology, Third Faculty of Medicine, Charles University, Ruská 87, 10000 Prague, Czech Republic; jan.pala@lf3.cuni.cz
[4] Department of Experimental Neurobiology, National Institute of Mental Health, Topolova 748, 25067 Klecany, Czech Republic; viera.kutna@nudz.cz
* Correspondence: Michael.hudec@lf3.cuni.cz

Supplementary materials

Table S1. Genetic and non-genetic risk factors associated with celiacdisease [1-3].

Genetic Factors Associated with Celiac Disease
Class II HLA alleles DQ2/8
Genetic disorders, Down and Turner's syndrome
**Non-Genetic Factors Associated with Celiac Disease**
First-degree relatives
Duration of gluten exposure
Age at gluten introduction
Duration of breast-feeding
Autoimmune disorders (Type 1 diabetes, Thyroid disease, hepatitis)
Anemia
Osteopenia disease
Skin disorders (Dermatitis herpetiformis)
Neurological disorders (Ataxia, Seizures, Myasthenia gravis)

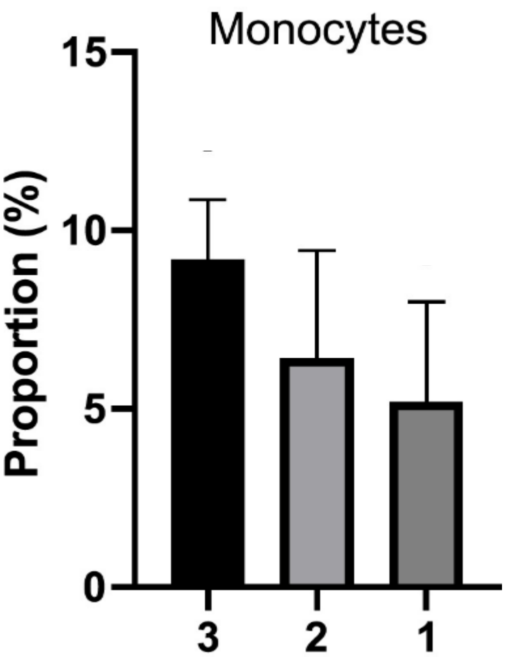

Figure S1. Supporting Figure 1. No statistically significant difference was found in the proportion of monocytes isolated from members of various familial generations(1-grandparents, 2-parents and 3-children).

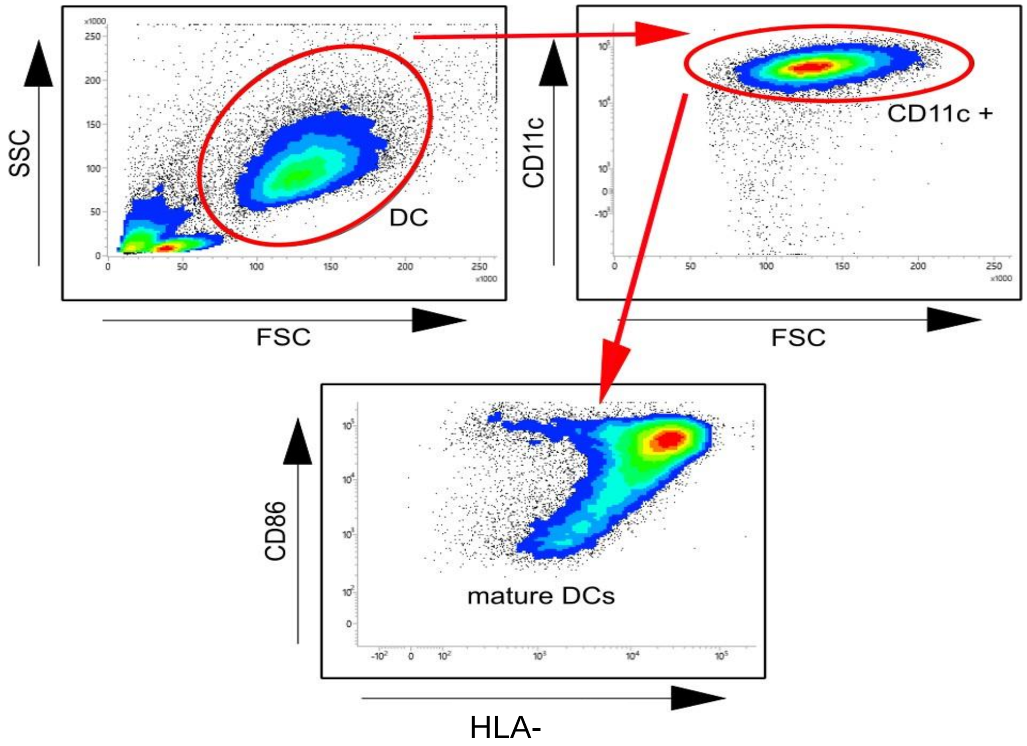

Figure S2. Supporting Figure 2. Representative example of the gating strategy for MoDC determination. MoDCs were determined by SSC/ FSC dot plot (upper left panel). The MoDC population was subsequently selected for the expression of lineage. Marker CD11c (upper right panel). The LPS/PTG activated MoDC population was controlledby the expression of maturation marker CD86 and antigen-presenting molecule HLA-DQ (lower panel).

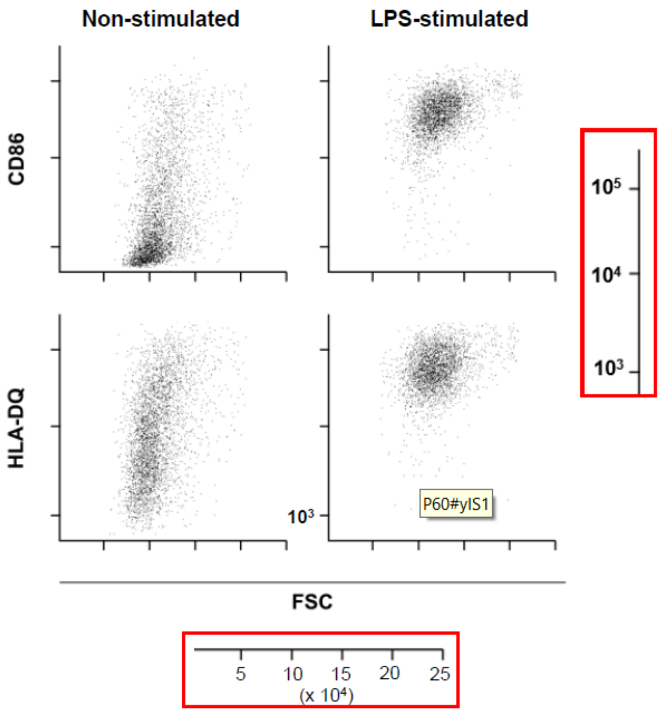

Figure S3. Supporting Figure 2 and 3. Representative forward scatter (FSC) dot plots of non-stimulated and LPS stimulated monocyte derived dendritic cells (MoDC) of healthy controls after 6 days cultivation, differentially expressing surface markers CD86 or HLA-DQ. Logarithmic scale provided (right).

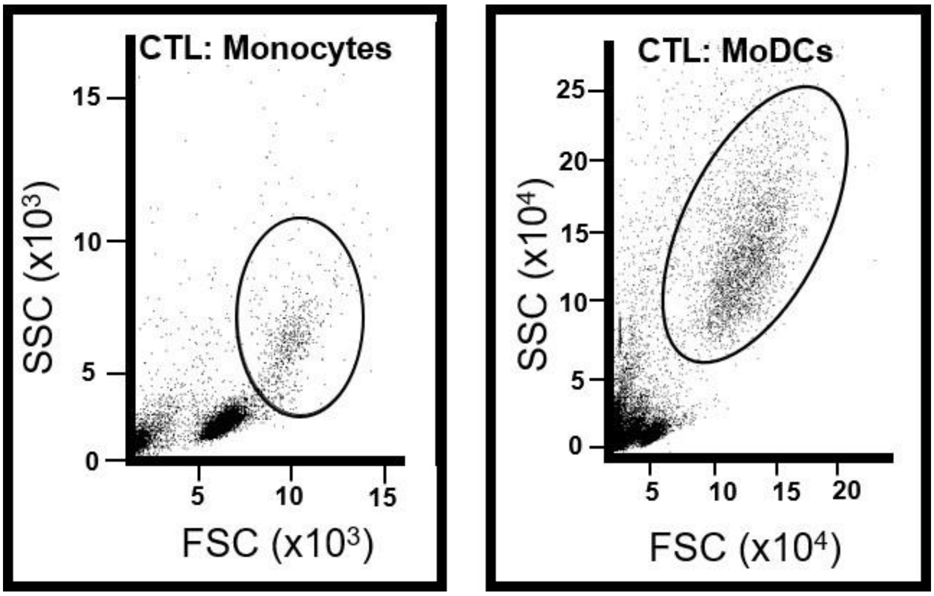

Figure S4. Supporting Figure 2. Dot plots indicating side scatter (SSC) versus forward scatter (FSC) profiles for monocytes (left) and stimulated monocyte derived dendritic cells (MoDCs; right) originating from healthy controls. Gating demarcates the percentage recovery of monocytes from peripheral mononuclear cells (left) or transformation efficiency to MoDCs post 24 hr exposure to LPS/PTG (right).

Monocyte Derived Dendritic Cells (MoDCs)		Median Fluorescence Intensity	
		CD86	HLA-DQ
LPS stimulated	Healthy Control	64,886	37,033
	Celiac Disease	52,184	25,075
PTG stimulated	Healthy Control	4,013	4,212
	Celiac Disease	46,666	27,911

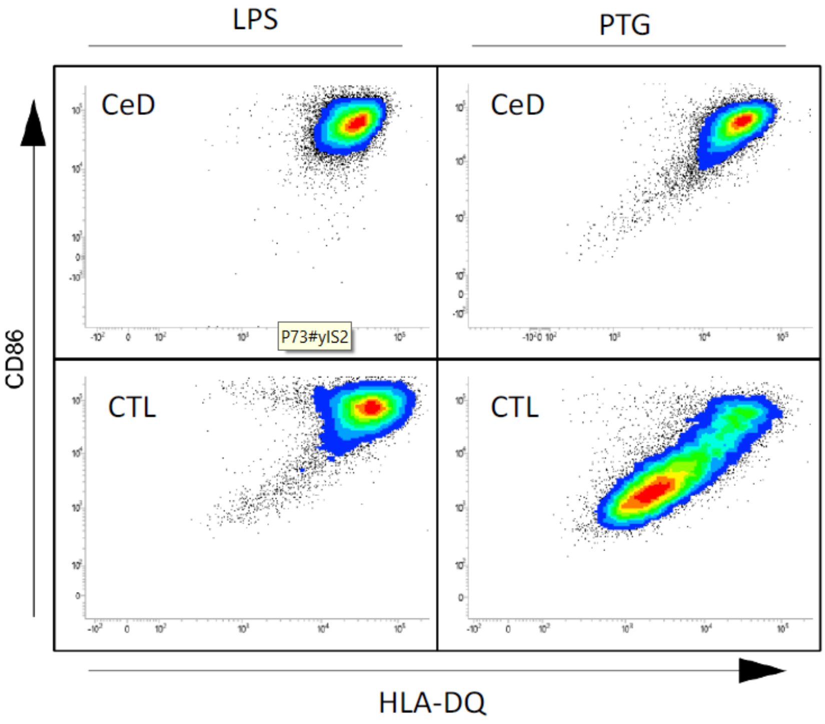

Figure S5. (Supporting Figure 3). Comparison of the flow cytometry data obtained following LPS or peptic-tryptic gliadin (PTG) fragment stimulation of MoDCs.MoDCs of CTLs stained by HLA-DQ (APC), CD86 (PE) show lower expression of selected markers after PTG stimulation. In contrast, MoDCs of CeD patients show similar reaction to LPS stimulation (Upper table provides exact MFI numbers obtained). Y axis represents CD86expression, X axis represents HLA-DQ expression. Lines represent donors of stimulated MoDCs (CD-upper, HC-lower) and columns represent stimulation factor (LPS-left, PTG-right).

References

1. Gujral, N., Freeman H.J., Thomson A.B., Celiac disease: prevalence, diagnosis, pathogenesis and treatment. *World J. Gastroenterol*. **2012**, *18*, 6036–59.
2. Ivarsson, A.; Hernell, O.; Stenlund, H.; Persson, L. Åke Breast-feeding protects against celiac disease. *Am. J. Clin. Nutr.* **2002**, *75*, 914–921, doi:10.1093/ajcn/75.5.914.
3. Goldsobel, A. Spector S. Allergy and immunology. *West J Med*. **1997**, *167*, 343.
